# Supplementary material for: SiCTeC: An inexpensive, easily assembled Peltier device for rapid temperature shifting during single-cell imaging
Source: PLoS Biol. 2020 Nov 6;18(11):e3000786. doi: 10.1371/journal.pbio.3000786 (PMC7685484; doi:10.1371/journal.pbio.3000786)
Supplement: S1 Table — The total bulk cost is $145.16, and the total itemized cost is $105.97 (excluding the optional plastic enclosure). All prices are in US dollars. SiCTeC, Single-Cell Temperature Controller. (DOCX) [file pbio.3000786.s001.docx]

| **Component** | **Price** | **Manufacturer (Supplier)** | **Part Number** |
| --- | --- | --- | --- |
| 12 V, 5 A power supply | $10.99 | LEDMO/EZON (Amazon) | 43207-895 |
| MD10C R3 motor driver | $11.50 | Cytron (Amazon) | MD10C |
| XY 3606 buck converter | $9.99 | Drok (Amazon) | 200217 |
| Solderable breadboard | $11.98 (3-pack) | Gikfun/Esohoo (Amazon) | 4330587492 |
| Micro Arduino | $19.40 | Arduino (Amazon) | ARD-A000059 |
| 10K potentiometer | $6.14 | Adafruit (Amazon) | 100511 |
| Buttons and resistors | $7.86 (assorted) | ELEGOO (Amazon) | EL-CK-000 |
| Peltier module | $28.30 | TE Technology | CH-38-1.0-0.8 |
| Silicone gasket | $30.00 (5-pack) | Grace Bio-Labs | JTR13R-A2-1.0 |
| NTC 3950 100K Thermistor | $9.00 (5-pack) | Happisland (Amazon) | CP-1004-01 |
| (Optional) Plastic enclosure | $12.99 | Pinfox (Amazon) | PF201275 |
