# Supplementary material for: SiCTeC: An inexpensive, easily assembled Peltier device for rapid temperature shifting during single-cell imaging
Source: PLoS Biol. 2020 Nov 6;18(11):e3000786. doi: 10.1371/journal.pbio.3000786 (PMC7685484; doi:10.1371/journal.pbio.3000786)
Supplement: S2 Table — The equation for PWM is as follows: PWM=Kpe(t)+∫Kie(t)dt+Kdde(t)dt, in which e(t) = Tsetpoint−T(t) is the temperature difference from the set point. PID, proportional-integral-derivative; PWM, pulse-width-modulation. (DOCX) [file pbio.3000786.s002.docx]

| ***K_p_*** | ***K_i_*** | ***K_d_*** |
| --- | --- | --- |
| 3 °C^-1^ | 0.03 °C^-1^ s^-1^ | 0.1 °C^-1^ s |
