# Supplementary figures and images for: SiCTeC: An inexpensive, easily assembled Peltier device for rapid temperature shifting during single-cell imaging
Source: PLoS Biol. 2020 Nov 6;18(11):e3000786. doi: 10.1371/journal.pbio.3000786 (PMC7685484; doi:10.1371/journal.pbio.3000786)

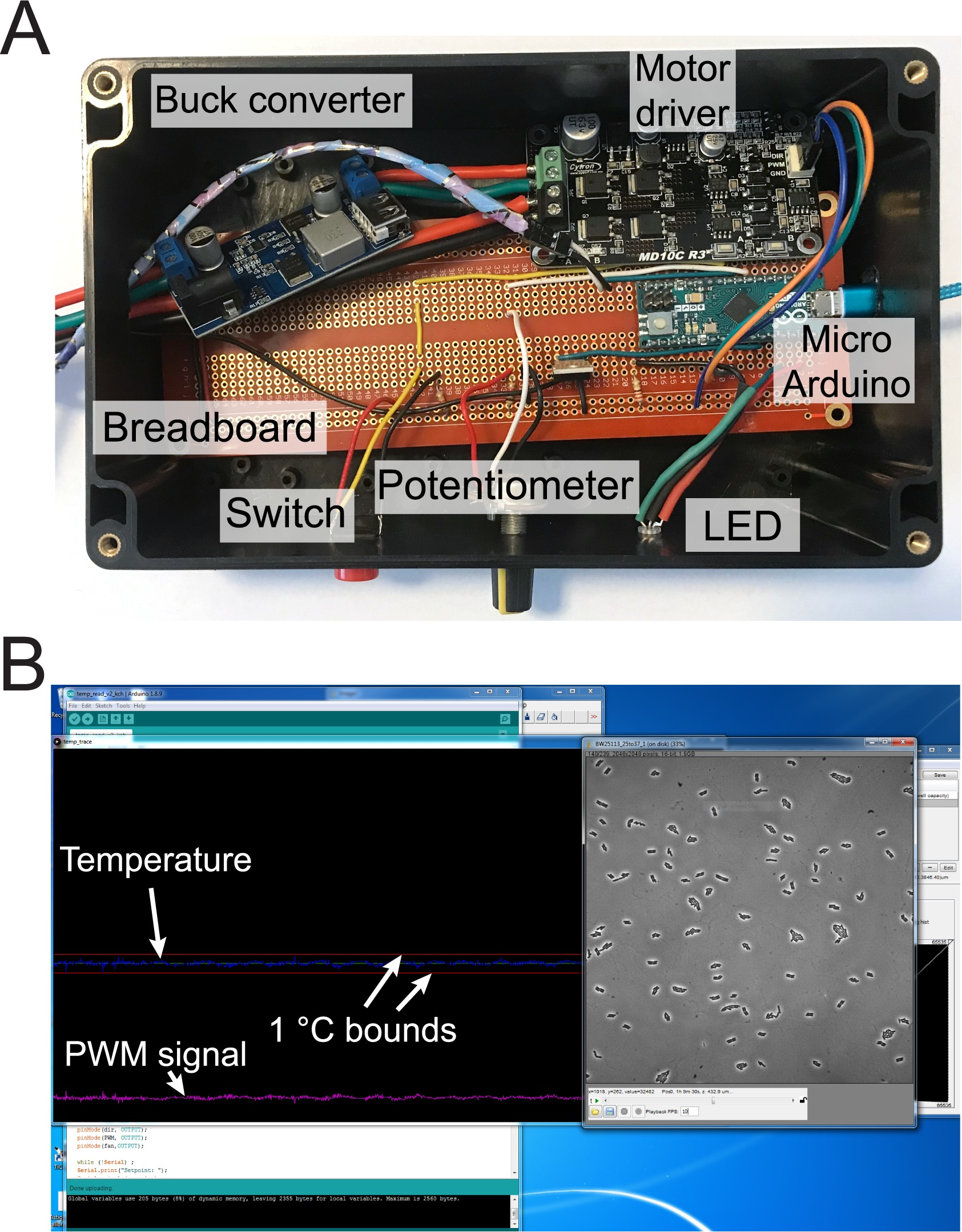

Supplement: S1 Fig — (A) Enclosed electrical components assembled into the temperature-control system. (B) Simultaneous imaging in μManager (right window) and temperature readout with the open-source software Processing (left window). SiCTeC, Single-Cell Temperature Controller. (TIF) [file pbio.3000786.s003.tif]

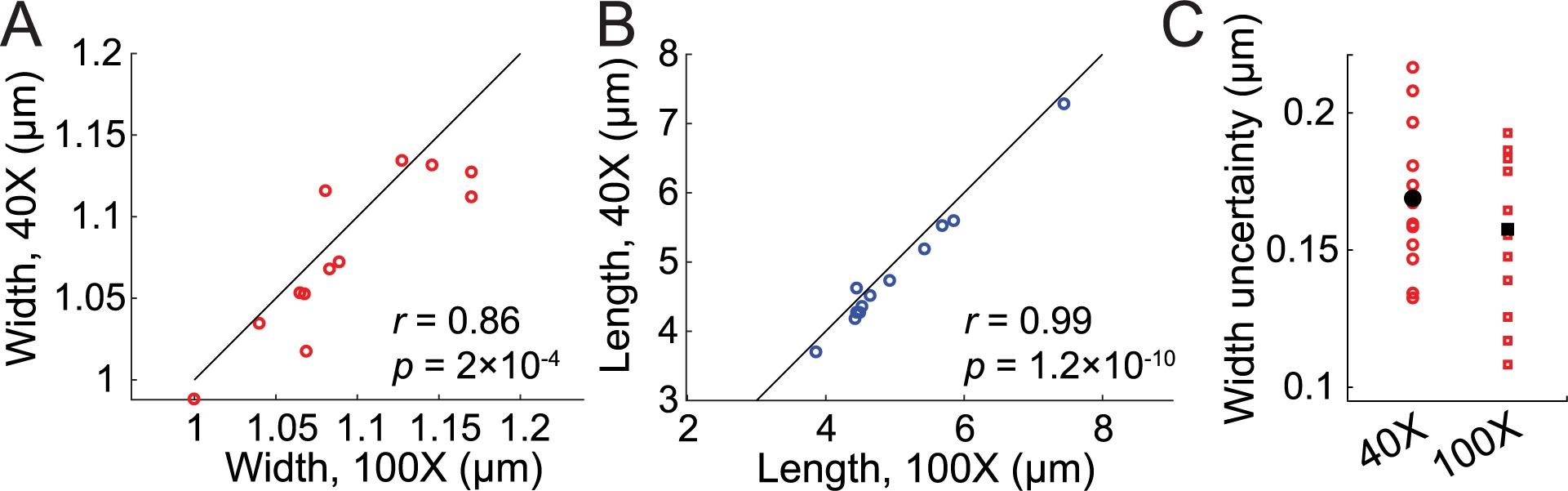

Supplement: S2 Fig — The same field of wild-type E. coli cells was imaged with 100X oil-immersion and 40X air objectives (n = 12 cells). (A) Measurements of cell width using 40X and 100X objectives were strongly correlated. Black line is y = x. r is Pearson’s correlation coefficient. The data underlying this figure can be found in file 11 in the Stanford Data Repository at https://purl.stanford.edu/cy453zg4308. (B) Measurements of cell length using 40X and 100X objectives were nearly perfectly correlated. Black line is y = x. r is Pearson’s correlation coefficient. The data underlying this figure can be found in file 11 in the Stanford Data Repository at https://purl.stanford.edu/cy453zg4308. (C) The uncertainty in individual width measurements with 40X and 100X objectives, defined as the mean absolute difference between the local width and the median width for each cell. Black-filled markers indicate mean values across cells. The data underlying this figure can be found in file 12 in the Stanford Data Repository at https://purl.stanford.edu/cy453zg4308. (TIF) [file pbio.3000786.s004.tif]

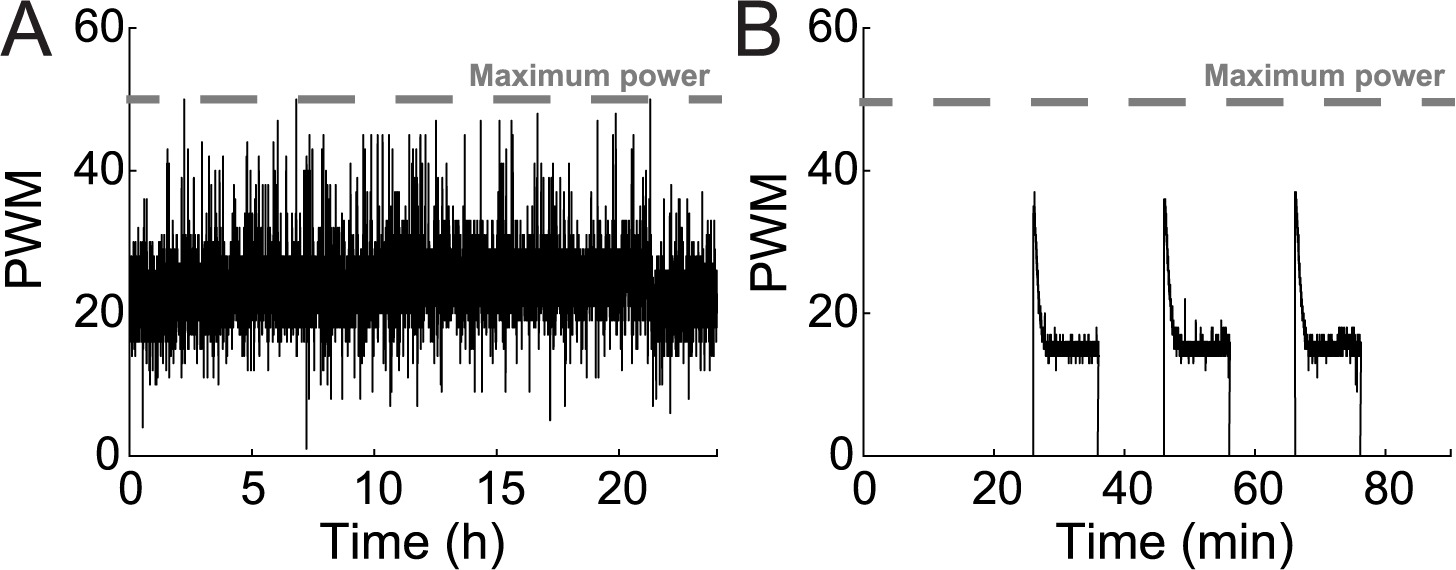

Supplement: S3 Fig — (A) The PWM signal during the 24-h experiment in which temperature was maintained at 37°C in Fig 3A. Ambient temperature was set to 25°C using the microscope temperature enclosure. The dashed line at PWM = 50 is the maximum signal delivered to SiCTeC (limited out of a possible 255). The data underlying this figure can be found in file 13 in the Stanford Data Repository at https://purl.stanford.edu/cy453zg4308. (B) The PWM signal during the experiment in which temperature was cycled between 30°C and 37°C in Fig 5A. Ambient temperature was set to 30°C (PWM = 0) using the microscope temperature enclosure. The data underlying this figure can be found in file 14 in the Stanford Data Repository at https://purl.stanford.edu/cy453zg4308. PWM, pulse-width-modulation; SiCTeC, Single-Cell Temperature Controller. (TIF) [file pbio.3000786.s005.tif]

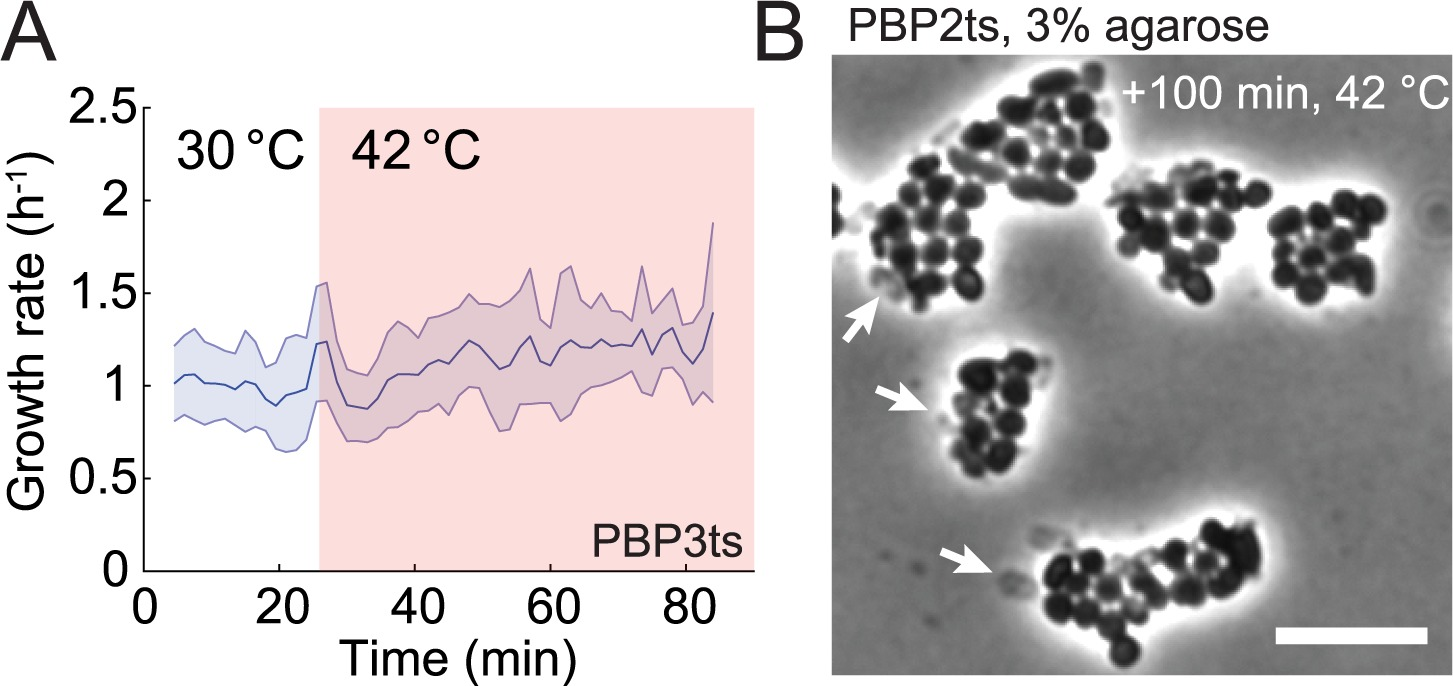

Supplement: S4 Fig — (A) PBP3ts cells maintained their growth rate throughout a temperature shift from 30°C to 42°C. Shaded error bars represent ±1 standard deviation (n = 102 cells). The data underlying this figure can be found in file 15 in the Stanford Data Repository at https://purl.stanford.edu/cy453zg4308. (B) After 100 min at 42°C, PBP2ts cells growing on 3% agarose pads were round or short rods. Arrows indicate lysed cells. Scale bar: 8 μm. PBP, penicillin-binding protein; ts, temperature sensitive. (TIF) [file pbio.3000786.s006.tif]
